# Our Contemporaries

**Published:** 1911-12

**Authors:** 


### OUR CONTEMPORARIES

*American Medicine,* October 1911, discusses editorially the work of the Erie County Medical Society (sic) and of the New York Academy of Medicine in regard to fee splitting and says that surgeons of surprisingly high standing are guilty. An apology is offered for not printing names—the discredit which would be brought to the profession.

*American Medicine* also calls attention to the relation of latitude to typhoid, suggesting that a fair comparison of different cities should discount this factor. However, we scarcely agree with the implication so far as a comparison of American and European or east and west coast American cities are concerned as the north-easterly trend of ocean currents counteracts about 10 degrees difference in latitude.

The *American Medical Journal* of St. Louis, (eclectic), October, 1911, notes rather sarcastically, the contemplated merger of the Barnes, P. & S. and American medical schools and foretells their destruction. As, last year, their combined registration was 274, it would seem that by joining forces, a good, strong college might result.

The same journal accuses the *Journal of the A.M.A.,* of stealing eclectic thunder in advocating the use of certain drugs which the eclectics have used for nearly a hundred years. But did not the plain, ordinary, unspecified physician whose successor is now termed allopathic by his opponents, use these same drugs a thousand years ago? The regular profession calls itself so with an apology for the implied superiority and counts among its numbers, the Indian medicine man, the African herb doctor, the mediaeval empiric, every practitioner of the healing art who has conformed to the standards of his time and environment and who has not voluntarily separated himself from the rank and file of his profession. If it comes to stealing thunder, how about that word *Eclectic?*

